# A giant new species of *Enchiridium* (Polycladida, Prosthiostomidae) from southwestern Japan

**DOI:** 10.3897/zookeys.918.47061

**Published:** 2020-03-12

**Authors:** Aoi Tsuyuki, Hiroshi Kajihara

**Affiliations:** 1 Graduate School of Science, Hokkaido University, Sapporo, Hokkaido 060-0810, Japan Hokkaido University Sapporo Japan; 2 Faculty of Science, Hokkaido University, Sapporo, Hokkaido 060-0810, Japan Hokkaido University Sapporo Japan

**Keywords:** Cotylea, marine flatworm, phylogeny, Platyhelminthes, taxonomy

## Abstract

We describe a new species of polyclad flatworm, *Enchiridium daidai***sp. nov.**, from the rocky subtidal zone in the East China Sea along the coasts of the Kyushu and Okinawa Islands, Japan. *Enchiridium daidai***sp. nov.** is characterized by i) the entire periphery of the dorsal surface narrowly fringed with orange, ii) a marginal-eyespot band extending to the position of the mouth (about anterior one-eighth of body), and iii) two prostatic vesicles covered by a common muscle sheath, which is penetrated by the ejaculatory duct. We performed a molecular phylogenetic analysis based on 945-bp 28S rDNA sequences of 16 species of Prosthiostomidae currently available in public databases in addition to those of *E. daidai***sp. nov.** and *Prosthiostomum torquatum* Tsuyuki et al., 2019. In the resulting tree, our new species was nested in a clade composed of *Enchiridium* species. The tree topology was in favor of a taxonomic view that *Enchiridium* should be defined by having i) a common muscle sheath that encloses two prostatic vesicles and ii) marginal eyespots that may or may not surround the periphery of the dorsal surface.

## Introduction

Polyclad flatworms in the family Prosthiostomidae Lang, 1884 are characterized by i) an elongated body with a ventral sucker after the female gonopore, ii) a plicate tubular pharynx, and iii) paired prostatic ducts, each of which extends from a spherical prostatic vesicle and enters the penis or the ejaculatory duct independently, instead of uniting to each other before the entrance. Prosthiostomidae is composed of five genera: *Enchiridium* Bock, 1913; *Enterogonimus* Hallez, 1913; *Euprosthiostomum* Bock, 1925; *Lurymare* Du Bois-Reymond Marcus & Marcus, 1968; and *Prosthiostomum* Quatrefages, 1845 (Faubel 1984; Litvaitis et al. 2019). The genus *Enchiridiumsensu*Faubel (1984) contains eight species: *E. delicatum* (Palombi, 1939); *E. evelinae* Marcus, 1949; *E. gabriellae* (Marcus, 1949); *E. japonicum* Kato, 1943; *E. magec*Cuadrado et al., 2017; *E. periommatum* Bock, 1913; *E. punctatum* Hyman, 1953; and *E. russoi* (Palombi, 1939). Members of this genus are distinguished from other prosthiostomids by having a muscle sheath (or bulb) that encloses just the two prostatic vesicles among other male reproductive organs; i.e., the seminal vesicle and the male atrium are not enclosed by the muscle sheath (Faubel 1984).

In Japan, 21 species of prosthiostomids were previously reported, but there was no record of *Enchiridium* (Kato 1944; Tsuyuki et al. 2019). In this paper, we describe a new species of *Enchiridium* from Kagoshima and Okinawa, Japan, based on morphological and molecular data. In addition, we infer the phylogenetic position of the new species within Prosthiostomidae from an analysis using partial 28S ribosomal DNA (28S rDNA) sequences.

## Material and methods

**Figure 1.**
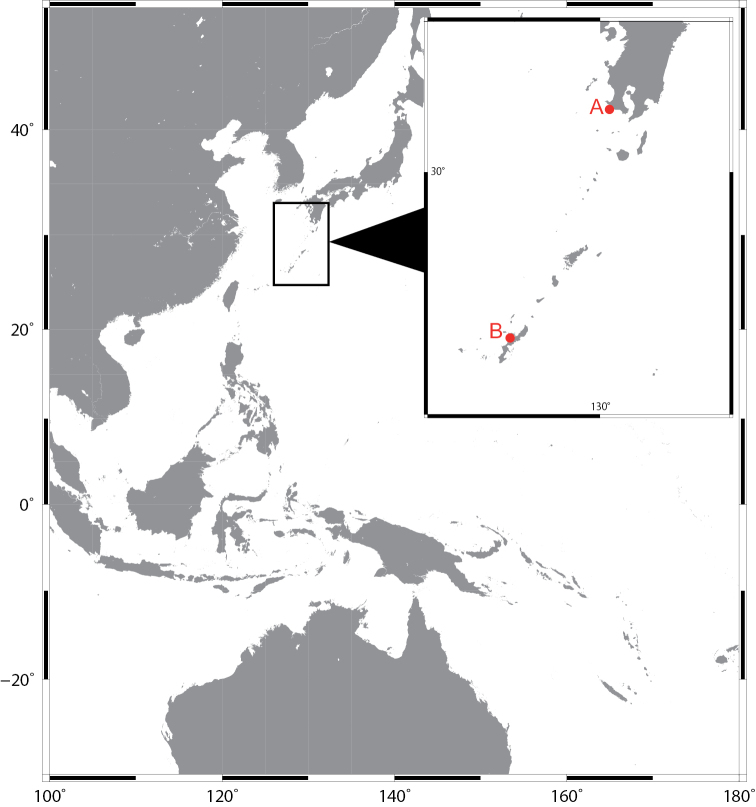
Map showing distribution of *Enchiridium daidai* sp. nov.: point A, off the coast of Bonomisaki, Kagoshima (type locality); point B, Nago, Okinawa Island.

Three polyclad specimens were collected subtidally from under rocks in Kagoshima and Okinawa, southwestern Japan (Fig. 1). Worms were anaesthetized in seawater containing menthol before fixation. The relaxed worms were photographed with a Nikon D5600 digital camera with external strobe lighting provided by a pair of Morris Hikaru Komachi Di flash units. For DNA extraction, a posterior piece of the body was removed and stored in 99.5% ethanol. The rest of the body was fixed in Bouin’s solution for 24 h and preserved in 70% ethanol for long-term storage.

For histological examination, tissues were dehydrated in an ethanol series, cleared in xylene, embedded in paraffin wax, and sectioned at a thickness of 7 µm using a microtome. Sections were stained with hematoxylin and eosin, mounted on glass slides in Entellan New (Merck, Germany), and then observed and photographed under an Olympus BX51 compound microscope. All type slides have been deposited in the Invertebrate Collection of the Hokkaido University Museum (ICHUM), Sapporo, Japan.

Total DNA was extracted using a silica-based method (Boom et al. 1990) after specimens were homogenized. A fragment (585 bp) of the cytochrome *c* oxidase subunit I (COI) gene was amplified with the primers Pros_COIF (5'-AGGTGTTTGAGCAGGTTTTATAGGTACAGG-3') and Pros_COIR (5'-ATGGGATCTCCTCCTCCTGAAGGRTC-3') for investigating intraspecific genetic distances. PerlPrimer ver. 1.1.21 (Marshall 2003–2011) was used to design these universal primers for Prosthiostomidae*de novo*, based on complete mitochondrial genome sequences (Aguado et al. 2016) from two prosthiostomids, *Enchiridium* sp. (GenBank KT363734) and *Prosthiostomum siphunculus* (Delle Chiaje, 1822) (GenBank KT363736). A 1017-bp fragment of 28S rDNA was amplified with the primers fw1 and rev2 (Sonnenberg et al. 2007) for molecular phylogenetic analyses. Polymerase chain reaction (PCR) amplification conditions were 94 °C for 5 min; 35 cycles of 94 °C for 30 s, 50 °C (COI) or 52.5 °C (28S rDNA) for 30 s, 72 °C for 1.5 min (COI) or 2 min (28S rDNA); and 72 °C for 7 min. All nucleotide sequences were determined by direct sequencing with a BigDye Terminator Kit ver. 3.1 and a 3730 Genetic Analyzer (Life Technologies, California, USA); two internal primers, hrms_fw2 (Oya et al. unpublished) and rev4 (Sonnenberg et al. 2007), were used in addition to fw1 and rev2 due to failure in sequencing by the internal primer fw2. Sequences were checked and edited by using MEGA ver. 7.0 (Kumar et al. 2016). In addition to three specimens collected in this study, a 946-bp partial sequence of the 28S rDNA from the holotype specimen of *Prosthiostomum torquatum* Tsuyuki et al., 2019 (ICHUM 5563) was determined by the same methods described above. All the edited sequences have been deposited in DDBJ/EMBL/GenBank.

Additional 28S rDNA sequences were downloaded from GenBank; *Pseudobiceros splendidus* (Lang, 1884) (Pseudocerotidae) and *Prostheceraeus crozieri* (Hyman, 1939) (Euryleptidae) were chosen as outgroups (Table 1). Sequences were aligned using MAFFT ver. 7.427 (Katoh et al. 2017), with L-INS-i strategy selected by the “Auto” option. Ambiguous sites were removed with Gblocks ver. 0.91b (Castresana 2002) using options for a less stringent selection. The optimal substitution model selected with MEGA ver. 7.0 (Kumar et al. 2016) under the Akaike Information Criterion (AIC) (Akaike 1974) was GTR+I+G. Phylogenetic analysis was performed with the maximum likelihood (ML) method by using RAxML ver. 8.2.10 (Stamatakis 2014). Nodal support within the ML tree was assessed by analyses of 1000 bootstrap pseudoreplicates (Felsenstein 1985). COI uncorrected *p*-distances were calculated using MEGA ver. 7.0 (Kumar et al. 2016).

All graphical treatments were done with Adobe Photoshop CC. Illustrations were prepared with Adobe Illustrator CC.

**Table 1.** List of species used for the molecular phylogenetic analysis and respective GenBank accession numbers.

Species	GenBank accession number	Reference
Prosthiostomidae		
*Enchiridium daidai* sp. nov.	LC504235	This study
	LC504236	
	LC504237	
*Enchiridium evelinae* Marcus, 1949	KY263683	Bahia et al. (2017)
*Enchiridium japonicum* Kato, 1943	MH700298	Litvaitis et al. (2019)
*Enchiridium periommatum* Bock, 1913	MH700299	Litvaitis et al. (2019)
	MH700300	
	MH700301	
*Enchiridium* sp. 1	MH700302	Litvaitis et al. (2019)
	MH700303	
*Enchiridium* sp. 2	MN384686	Dittmann et al. (2019)
*Enchiridium* sp. 3	KY263673	Bahia et al. (2017)
*Enchiridium* sp. 4	KY263679	Bahia et al. (2017)
*Euprosthiostomum mortenseni* Marcus, 1948	MH700304	Litvaitis et al. (2019)
*Prosthiostomum acroporae* (Rawlinson et al., 2011)	HQ659011	Rawlinson et al. (2011)
*Prosthiostomum cynarium* Marcus, 1950	MH700371	Litvaitis et al. (2019)
*Prosthiostomum lobatum* Pearse, 1938	MH700372	Litvaitis et al. (2019)
*Prosthiostomum milcum* Du Bois-Reymond Marcus & Marcus, 1968	MH700373	Litvaitis et al. (2019)
*Prosthiostomum purum* Kato, 1937	MH700374	Litvaitis et al. (2019)
*Prosthiostomum siphunculus* (Delle Chiaje, 1822)	HQ659012	Rawlinson et al. (2011)
*Prosthiostomum torquatum* Tsuyuki et al., 2019	LC504234	This study
*Prosthiostomum trilineatum* Yeri & Kaburaki, 1920	MH700376	Litvaitis et al. (2019)
*Prosthiostomum utarum* Marcus, 1952	MH700377	Litvaitis et al. (2019)
*Prosthiostomum* sp.	MH700375	Litvaitis et al. (2019)
Outgroup		
*Prostheceraeus crozieri* (Hyman, 1939)	HQ659013	Rawlinson et al. (2011)
*Pseudobiceros splendidus* (Lang, 1884)	MH700388	Litvaitis et al. (2019)

## Results

### Family Prosthiostomidae Lang, 1884

**Genus *Enchiridium* Bock, 1913 *sensu*Faubel (1984)**

#### Enchiridium daidai sp. nov.

Taxon classificationAnimaliaPolycladidaProsthiostomidae

D68F7767-8135-5CC8-BC14-B8E5757DF66D

http://zoobank.org/5D0FCB54-F262-4616-8790-FD210878679E

**Figure 2.**
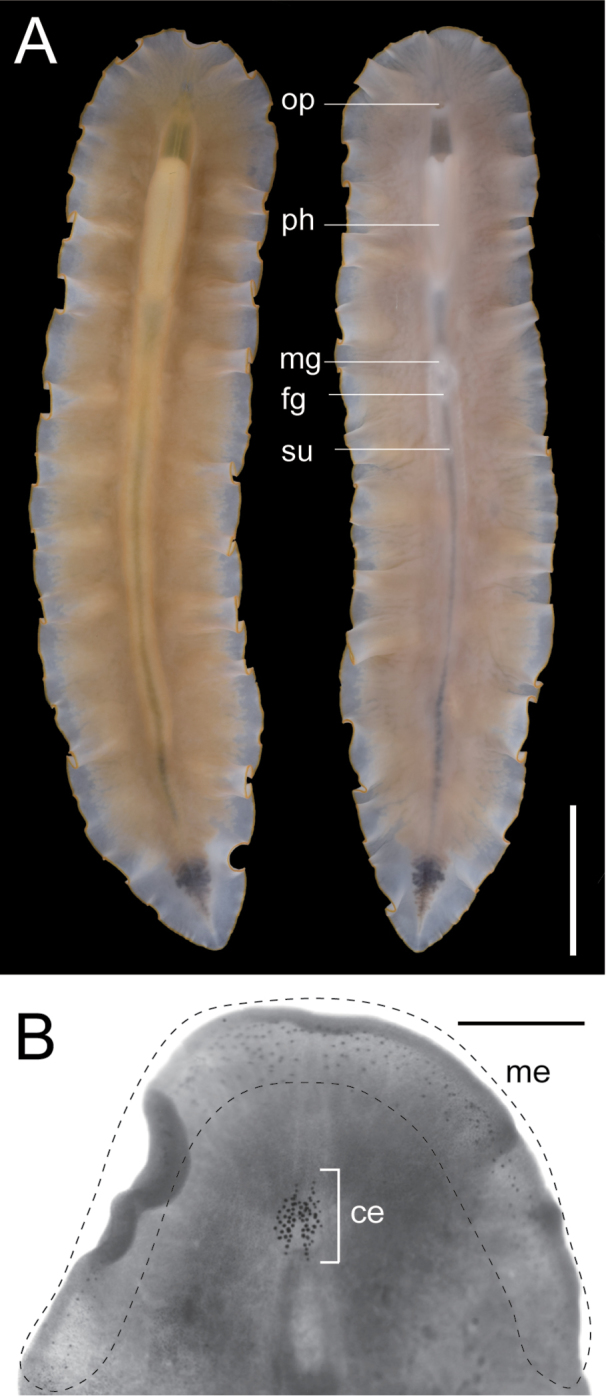
*Enchiridium daidai* sp. nov., photograph taken in life and eyespots observed in fixed state after being cleared in xylene. **A**ICHUM 5993 (holotype), entire animal, dorsal view (left) and ventral view (right) **B**ICHUM 5994 (paratype), magnification of anterior body, showing arrangements of cerebral and marginal eyespots. Abbreviations: **ce** cerebral eyespots **fg** female gonopore **me** marginal eyespots **mg** male gonopore **op** oral pore **ph** pharynx **su** sucker. Scale bars: 10 mm (**A**); 1 mm (**B**).

**Figure 3.**
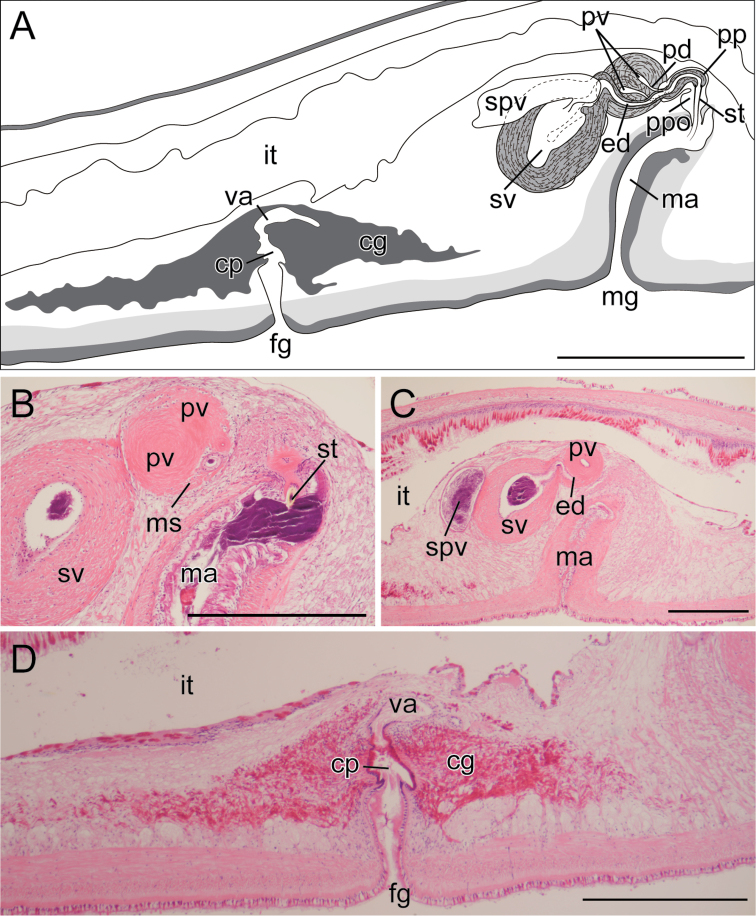
*Enchiridium daidai* sp. nov., ICHUM 5993 (holotype), schematic diagram (**A**) and sagittal sections (**B–D**), anterior to the right. **A** Schematic diagram of copulatory complex **B** a common muscle sheath/bulb enclosing two prostatic vesicles and penis stylet **C** ejaculatory duct penetrating a common muscle sheath/bulb **D** female copulatory apparatus. Abbreviations: **cg** cement glands **cp** cement pouch **ed** ejaculatory duct **fg** female gonopore **it** intestine **ma** male atrium **mg** male gonopore **ms** muscle sheath/bulb **pd** prostatic duct **pp** penis papilla **ppo** penis pouch **pv** prostatic vesicle **spv** spermiducal vesicle **st** stylet **sv** seminal vesicle **va** vagina. Scale bars: 500 µm.

**Figure 4.**
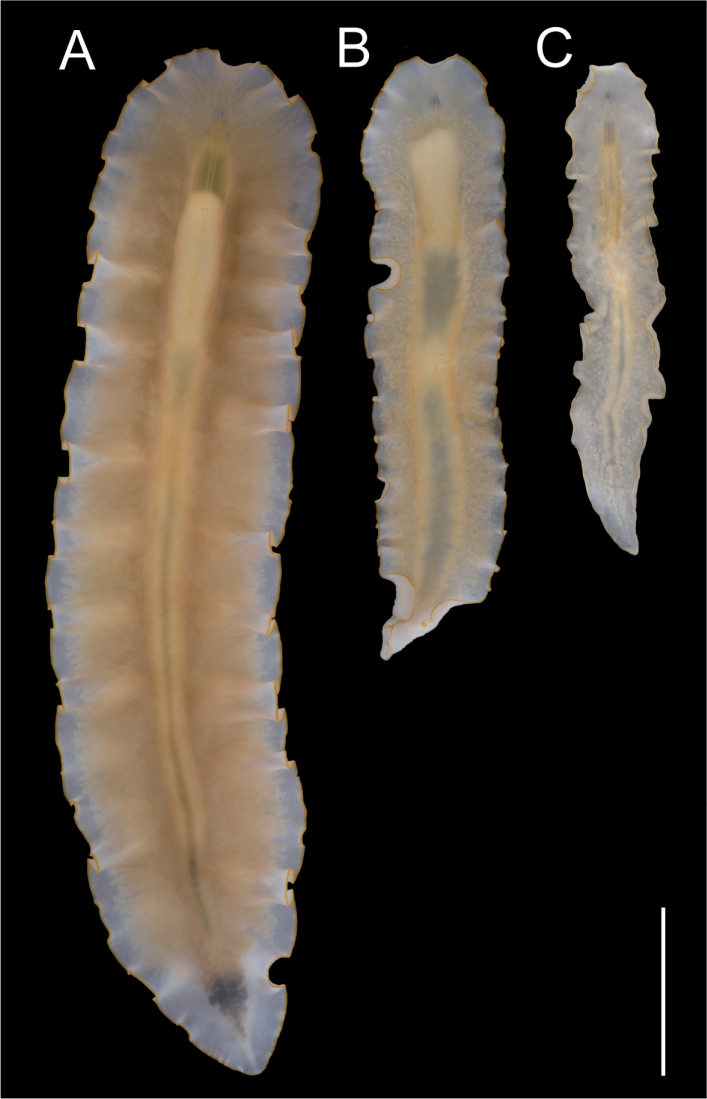
Difference in mature body size among *Enchiridium daidai* sp. nov. **A**ICHUM 5993 (holotype), from Kagoshima **B**ICHUM 5995 (paratype), from Okinawa **C**ICHUM 5994 (paratype), from Okinawa. Scale bar: 10 mm.

New Japanese name: daidai-hoso-hiramushi Figures 2 , 3 , 4

##### Etymology.

The new specific name *daidai* is a Japanese noun, meaning the color orange. It was named after the thin marginal orange line surrounding the entire dorsal fringe.

##### Material examined.

Three specimens, all collected by A. Tsuyuki. ***Holotype***: ICHUM 5993, sagittal sections through reproductive structures (22 slides), and the rest of the body, unsectioned, preserved in 70% ethanol, collected at 13–14 m depth off the coast of Bonomisaki (31.2542N, 130.2150E), Kagoshima, Japan, on 26 July 2018. ***Paratypes***: ICHUM 5994, sagittal sections through head to reproductive structures (9 slides); ICHUM 5995, cross sections through reproductive structures (21 slides); both collected at 5 m depth in Nago (26.6013N, 127.9137E), Okinawa, Japan, on 22 May 2019.

##### Type locality.

Off the coast of Bonomisaki (31.2542N, 130.2150E), Kagoshima, Japan.

##### Description.

Body elongated, tapered posteriorly, 28–77 mm long (77 mm in holotype) and 4.6–14 mm maximum width (14 mm in holotype) in living state (Fig. 2A); anterior margin rounded; mid-point of posterior margin acute. Tentacles absent. Dorsal surface smooth, translucent, fringed with thin marginal orange line (Fig. 2A). Ventral surface translucent, without color pattern. Pair of cerebral-eyespot clusters, each consisting of 20–52 eyespots (left 20 and right 23 in holotype); each cluster of an antero-posteriorly elongated spindle shape (Fig. 2B). Marginal-eyespot clusters forming single marginal band, extending to position of mouth (about anterior one-eighth of body) along margins on both sides; marginal eyespots abundant along anterior margin, diminishing posteriorly (Fig. 2B). Ventral eyespots absent. Intestine highly branched, spreading all over body. Plicated pharynx tubular in shape, about one-fifth of body length, located in anterior one-third of body (Fig. 2A). Oral pore situated at anterior end of pharynx, behind brain. Male gonopore and female gonopore closely set, both situated behind posterior end of pharynx. Male copulatory apparatus consisting of large seminal vesicle, pair of prostatic vesicles, and armed penis papilla (Fig. 3A). Antero-posterior length of seminal vesicle more than twice as long as diameter of each prostatic vesicle. Spermiducal vesicles forming single row on each side of midline, separately entering into seminal vesicle. Ejaculatory duct with thick muscular layer, entering penis papilla. Prostatic ducts with muscular layer, connected to ejaculatory duct separately at proximal end of penis papilla. Pair of spherical prostatic vesicles coated within thin non-nucleated muscular wall, arranged anterodorsally to ejaculatory duct. Common muscular sheath enclosing two prostatic vesicles (Fig. 3B). Seminal vesicle oval, coated with thick muscular wall, narrowing anteriorly and forming ejaculatory duct; latter almost immediately penetrating common muscular sheath (Fig. 3C). Penis papilla armed with pointed tubular stylet, enclosed in penis pouch, protruding into male atrium (Fig. 3C). Male atrium elongated anteriorly, lined with ciliated, muscularized epithelium (Fig. 3B). Female reproductive system immediately posterior to male reproductive system. Cement glands numerous, concentrated around vagina and releasing their contents in cement pouch (Fig. 3D). Vagina curving anteriorly, leading to two narrow lateral branches of uteri. Each branch of uteri turning laterally and then running backwards. Lang’s vesicle absent. Sucker set on body center (Fig. 2A).

##### Habitat.

Subtidal (5–14 m depth), under rocks.

##### Variation.

Specimens from Kagoshima and Okinawa differed in body size. The holotype from Kagoshima was 77 mm long and 15 mm wide, whereas the paratype specimens from Okinawa were 28–37 mm long and 4.6–7.4 mm wide (Fig. 4).

##### Diagnosis.

Body elongated, usually rounded anteriorly; dorsal surface translucent, fringed by a thin marginal orange line; marginal eyespots present only anteriorly; plicated pharynx tubular in shape, about one-fifth of body length; pair of prostatic vesicles bound by common muscular sheath, the latter penetrated by ejaculatory duct.

##### Sequences.

Partial COI (585 bp) and 28S rDNA (1017 bp) sequences from three individuals: LC504240 (COI), LC504235 (28S rDNA) from ICHUM 5993 (holotype); LC504238 (COI), LC504236 (28S rDNA) from ICHUM 5994 (paratype); LC504239 (COI), LC504237 (28S rDNA) from ICHUM 5995 (paratype).

##### Molecular phylogeny and genetic distances.

**Figure 5.**
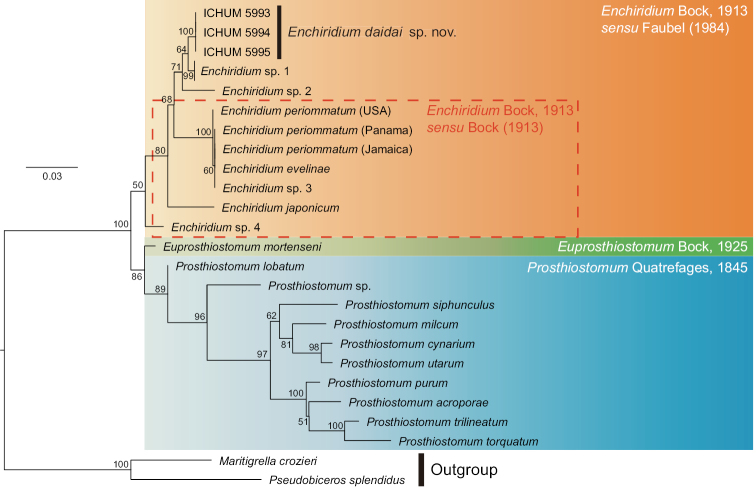
Maximum likelihood phylogenetic tree based on 935-bp 28S rDNA. Bootstrap support values are indicated near nodes.

In the phylogenetic tree, *Enchiridium daidai* sp. nov. was nested in a clade composed of *Enchiridium* species (Fig. 5). The genetic distances (uncorrected *p*-values) for the COI sequences among three specimens of *Enchiridium daidai* sp. nov. were 0.002–0.012. Genetic distances between individuals from different localities (Kagoshima vs. Okinawa), 0.010–0.012, were larger than that between individuals from the same locality (Okinawa), 0.002.

##### Remarks.

In spite of the noticeable difference in body size, specimens from Kagoshima and Okinawa – all having reached sexual maturity – were identified as conspecific. They shared the following morphological characteristics: i) body dorsally fringed with a thin orange line, ii) marginal-eyespot band extending to the position of the mouth (about anterior one-eighth of the body), iii) two prostatic vesicles covered by a common muscle sheath, and iv) common muscle sheath penetrated by ejaculatory duct. In addition, the COI*p*-distances among the specimens, 0.002–0.012, fell in a range of intraspecific values, 0.000–0.020, which was observed in four species of the acotylean leptoplanoid *Notocomplana* (Oya and Kajihara 2017), thus rendering support for our interpretation of conspecificity. Within Polycladida, remarkable intraspecific variation in body size has been reported for the acotylean stylochoid *Planocera reticulata* (Stimpson, 1855), which was recorded to vary by 10–80 mm in length and 6–45 mm in width (Yeri and Kaburaki 1918). Among the cotylean Proshiostomidae, sexually matured individuals of *Prosthiostomum cyclops* (Verrill, 1901) have been reported to vary a great deal (> ×10) in size by locality: 75–90 mm long × 10–15 mm wide in the Bermuda Islands (Verrill 1901), whereas 6.5 mm long × 1.7 mm wide in the islands of Bonaire and Klein Bonaire (Du Bois-Reymond Marcus and Marcus 1968). These observations may imply that these polyclads undergo an indeterminate growth, in which growth is not terminated after reaching adulthood, although other factors – such as geographical and ecological ones – must also be taken into account.

As for the taxon concept of *Enchiridium*, our results did not show a compatibility to Bock’s (1913) original view on the genus. The genus *Enchiridium* was established by Bock (1913) for *E. periommatum* based on two characteristics: i) two prostatic vesicles enclosed in a common muscle sheath, and ii) marginal eyespots completely surrounding the entire periphery of the dorsal surface. Subsequently, *E. evelinae*, *E. japonicum*, and *E. punctatum* were added to the genus (Kato 1943; Marcus 1949; Hyman 1953) before Faubel (1984) re-defined *Enchiridium*. It was circumscribed so that “only the prostatic vesicles are bound into a common muscle bulb and oriented anterodorsal to the ejaculatory duct” (Faubel 1984, p. 231); namely, the encircling marginal eyespots were not regarded as a necessary condition for *Enchiridium*. At the same time, Faubel (1984) transferred three *Lurymare* species, *viz.*, *L. delicatum*, *L. gabriellae*, and *L. russoi*, into *Enchiridium*. As a result, seven species were included in *Enchiridium* in the taxonomic system of Faubel (1984). In contrast, Prudhoe (1985) supported Bock’s (1913) taxon concept of *Enchiridium*, retaining four species, *E. evelinae*, *E. japonicum*, *E. periommatum*, and *E. punctatum*, in *Enchiridium* and three species, *L. delicatum*, *L. gabriellae*, and *L. russoi*, in *Lurymare*. On the other hand, Cuadrado et al. (2017) followed Faubel’s (1984) redefinition when they established *E. magec*. The monophyly of *Enchidirium* sensu Faubel (1984) was strongly supported in a molecular phylogenetic analysis based on partial 28S rDNA (Litvaitis et al. 2019). In our study, *Enchidiriumsensu*Faubel (1984) received 80% bootstrap support with the exclusion of *Enchiridium* sp. 4 of Bahia et al. (2017); including the latter, the branch support decreased to 50% (Fig. 5). Also, *Enchiridium* in the sense of Bock (1913) and Prudhoe (1985) – represented by *E. evelinae*, *E. japonicum*, *E. periommatum*, *Enchiridium* sp. 3, and *Enchiridium* sp. 4 (cf. Bahia et al. 2017, Table 2) in our analysis – was not monophyletic. Therefore, the taxonomy of *Enchiridium* should be revised with further molecular phylogenetic analyses as well as careful examination of morphological characters among the constituent members. At the moment, however, we adopt Faubel’s (1984) redefinition and place our new species in the genus *Enchiridium* along with eight other species. We did so because our results indicated that the arrangement of the marginal eyespots should not be taken into account as generic diagnostic characters.

*Enchiridium daidai* sp. nov. is distinguished from *E. evelinae*, *E. japonicum*, *E. periommatum*, and *E. punctatum* by the arrangement of the marginal eyespots; the marginal-eyespot band in these four species completely encircles the periphery of the dorsal surface, whereas that of our specimens is present only along the anterior margin. Our new species is also easily distinguished from the other four congeners, *E. delicatum*, *E. gabriellae*, *E. magec*, and *E. russoi*, by the thin marginal orange line surrounding the entire dorsal fringe and by the lack of spots or maculae on the dorsal surface (Table 2).

Reaching 77 mm in body length, *Enchiridium daidai* sp. nov. is the largest species in the genus over *E. punctatum* (about 40 mm in body length; Hyman 1953, p. 386). Indeed, *E. daidai* is the second largest in the Prosthiostomidae after *P. cyclops*, which reaches 90 mm (Verrill 1901). Among about 80 species of prosthiostomids, only *E. daidai* sp. nov. and *P. cyclops* are known to exceed 70 mm in body length, while most of the other species are less than 30 mm long. Therefore, our new species is considered to be unusually big in body size for a prosthiostomid.

**Table 2.** Comparison of characters between five *Enchiridium* species in which marginal eyespots are distributed only anteriorly.

	*E. daidai* sp. nov.	*E. delicatum*	*E. gabriellae*	*E. magec*	*E. russoi*
**Type locality**	Off the coast of Bonomisaki, Kagoshima, Japan	East London, South Africa	São Sebastião Island, São Paulo, Brazil	North of El Balito, Tenerife, Canary Islands, Spain	Shelley Beach, East London, South Africa
**Dorsal coloration/ pattern**:					
Background color	Translucent	Light pale yellow	Transparent	Whitish to cream	Greyish yellow
Spots or maculae on dorsal surface	None	None	None	Brown caramel spots, arranged more densely in the central region	Brown pigment spots spread especially in the central part
Median line	None	Two yellow bands	None	A band composed of brown caramel spots	An ocher yellow band
Fringed line	A thin orange line	None	None	None	None
**Reference**	This study	Palombi (1939)	Marcus (1949)	Cuadrado et al. (2017)	Palombi (1939)

## Supplementary Material

XML Treatment for Enchiridium daidai
